# Prevalence of inherited ichthyosis in France: a study using capture-recapture method

**DOI:** 10.1186/1750-1172-9-1

**Published:** 2014-01-06

**Authors:** Isabelle Dreyfus, Cécile Chouquet, Khaled Ezzedine, Sophie Henner, Christine Chiavérini, Aude Maza, Sandrine Pascal, Lauriane Rodriguez, Pierre Vabres, Ludovic Martin, Stéphanie Mallet, Sébastien Barbarot, Jérôme Dupuis, Juliette Mazereeuw-Hautier

**Affiliations:** 1 Reference Centre for Rare Skin Diseases, Dermatology Department, Larrey Hospital, CHU Toulouse, Toulouse, France; 2 Institut de Mathématiques de Toulouse, Laboratoire de Statistique et Probabilités, CNRS (UMR 5219), Paul Sabatier University, Toulouse, France; 3 Reference Centre for Rare Skin Diseases, Dermatology Department, CHU Bordeaux, Bordeaux, France; 4 Reference Centre for epidermolysis bullosa, Dermatology Department, CHU Nice, Nice, France; 5 Competence Centre for Rare Skin Diseases Dermatology Department, CHU Dijon, Dijon, France; 6 Dermatology Department, CHU Angers, Angers, France; 7 Dermatology Department, CHU Marseille, Marseille, France; 8 Competence Centre for Rare Skin Diseases Dermatology Department, CHU Nantes, Nantes, France

**Keywords:** Inherited ichthyosis, Epidemiology, Prevalence, Capture-recapture method, Genodermatosis

## Abstract

**Background:**

Inherited ichthyoses represent a group of rare skin disorders characterized by scaling, hyperkeratosis and inconstant erythema, involving most of the tegument. Epidemiology remains poorly described. This study aims to evaluate the prevalence of inherited ichthyosis (excluding very mild forms) and its different clinical forms in France.

**Methods:**

Capture – recapture method was used for this study. According to statistical requirements, 3 different lists (reference/competence centres, French association of patients with ichthyosis and internet network) were used to record such patients. The study was conducted in 5 areas during a closed period.

**Results:**

The prevalence was estimated at 13.3 per million people (/M) (CI95%, [10.9 – 17.6]). With regard to autosomal recessive congenital ichthyosis, the prevalence was estimated at 7/M (CI 95% [5.7 – 9.2]), with a prevalence of lamellar ichthyosis and congenital ichthyosiform erythroderma of 4.5/M (CI 95% [3.7 – 5.9]) and 1.9/M (CI 95% [1.6 – 2.6]), respectively. Prevalence of keratinopathic forms was estimated at 1.1/M (CI 95% [0.9 – 1.5]). Prevalence of syndromic forms (all clinical forms together) was estimated at 1.9/M (CI 95% [1.6 – 2.6]).

**Conclusions:**

Our results constitute a crucial basis to properly size the necessary health measures that are required to improve patient care and design further clinical studies.

## Background

Inherited ichthyoses represent a large group of monogenic Mendelian disorders of cornification due to mutations in genes involved in skin barrier function [1]. Skin changes, affecting all or most of the tegument, are characterized by scaling, hyperkeratosis or both. Erythema is inconstantly present. According to a recent ichthyosis expert consensus, the classification should be clinically based and distinguish between syndromic and non-syndromic ichthyoses [2].

Except for very mild forms of ichthyosis, the disease was demonstrated to have a significant impact on quality of life (QOL) [3-7]. During the past few years, much progress has been achieved in describing the molecular basis of these disorders and in establishing genotype-phenotype correlations [8]. However, prevalence remains poorly described [9-16] and no patient registration exists. Understanding epidemiology of rare skin disorders such as ichthyosis is one of the main objectives advocated in the *Plan Maladies Rares* defined by the French Health ministry [17].

The objective of our study was to estimate the prevalence of inherited ichthyoses having a significant impact on QOL (excluding accordingly very mild forms) and to determine the prevalence of their different clinical forms. Patients suffering from such inherited ichthyoses are primarily diagnosed by private or hospital physicians and subsequently usually followed in hospital. Nevertheless, a “hospital list” may not be exhaustive to enumerate such patients, since some of them, who could have been demotivated by the lack of effective treatment for example, might have interrupted their medical care and thus are “out of the hospital healthcare system”. It is therefore necessary to identify these patients *via* additional lists of recruitment, hence the choice of capture-recapture method [18-24].

Capture-recapture models are commonly used for estimating prevalence in epidemiological studies when information comes from incomplete lists [18,19]. Estimation is based on the number of cases recorded on each list, as well as the overlapping of cases between the lists. This thereby allows for estimation of the number of cases of the target population appearing on no list. The standard statistical approach uses log-linear models. They allow for dealing with possible dependencies between lists, but also include, in particular, the independent model which assumes independence between lists [20,21]. The implementation of such an approach requires at least 3 sources of data.

## Methods

The study protocol was reviewed and approved both by an independent health ethics committee in Toulouse, France and by the CNIL, the French National authority for registers.

The target population consisted of patients suffering from inherited ichthyosis with a confirmed diagnosis performed by a physician at a given time of their illness. We excluded very mild forms that can be defined as ichthyoses with a very mild clinical severity (visual analogue scales (VAS) for erythema and scaling < 1.5/10, in the absence of systemic therapy by acitretin). In practice, these very mild forms correspond to ichthyosis vulgaris or ichthyoses with similar severity.

According to statistical requirements, 3 lists of patients were selected: patients registered in reference/competence centres that are experts for rare skin diseases (L1), members of the French association of patients with ichthyosis (named AIF) (L2) and members of the Facebook social network: “*L’ichtyose qu’est-ce que c’est*?” (L3). The study was conducted in 5 French areas (Aquitaine, Bourgogne, Midi-Pyrénées, Pays de la Loire, Provence Alpes Côte d’Azur (PACA)) during a closed period of 6 weeks (from June 1^st^ 2011 to July 15^th^ 2011). Patients were contacted either by phone (L1, L2) or by email (L3) to obtain their consent. Overlaps between lists were identified by checking the first 3 letters of the surname, the first 2 letters of the first name and the date of birth. In order to confirm the population prerequisite closed in time and space, all patients were also asked about their zip code and whether they resided in the selected area for a minimum of 6 weeks (without migratory movements).

For the patients recruited *via* L1, the diagnosis of the clinical form of ichthyosis and the severity were determined by the clinician in charge. The patients recorded *via* L2 or L3 (without overlapping with L1) were asked to fill-in a questionnaire exploring ichthyosis characteristics (including VAS for erythema and scaling), which was then reviewed by a board of ichthyosis experts to define the clinical form and the severity (data not shown).

The number of patients suffering from ichthyosis was estimated by considering a set of log-linear models, taking into account possible interactions between lists. In addition to area, individual covariates - such as gender, age (older than 15 years or younger) and grade of severity (mild/moderate/severe/very severe) - were introduced in the model in order to improve the prevalence estimation [25]. A selection model procedure was implemented using the GENMOD procedure of SAS: the best model being the one with the maximum weighted Akaike Information Criterion (wAIC) [26]. The selected model allowed for estimation of the number of ichthyosis patients at several levels: estimation for each area and for the pooled areas, as well as estimation by age group and by severity. Estimated prevalence was finally deduced from the estimated number of ichthyosis patients and the French 2008 census: for each area (regional prevalence) and for the pooled areas (global prevalence), which can be considered as national prevalence, subject to representativeness of the selected areas.

With regard to clinical form, a separate *ad hoc* statistical procedure was implemented (clinical form cannot be introduced as covariate in the log-linear model because of a very low number of detected patients for some clinical forms). The prevalence of each clinical form was estimated by applying the distribution of clinical forms observed among the reported cases to the estimated global prevalence. This *ad hoc* procedure is based on the assumption that the capture probability depends on the clinical form mainly through the severity grade, which seems reasonable from a dermatological point of view.

## Results

### Three-source observed data

A total of 119 patients suffering from ichthyosis were identified. The observed frequencies according to presence or absence on each list for each area are presented in Table 1. Data was unbalanced: the majority of patients were identified *via* L1, whereas very few patients were recorded *via* L3. Overlaps were rather rare and mostly observed between L1 and L2. No patient was captured simultaneously in the 3 lists. Furthermore, some geographic disparities were observed concerning the recruitment of the patients.

**Table 1:** Numbers of patients reported in the three lists, in each of the 5 selected areas

**Area**	**100**^**a**^	**010**	**001**	**110**	**011**	**101**	**111**	**Total**
Aquitaine	5	5	1	3	0	0	0	14
Pays de Loire	10	3	2	5	0	0	0	20
Midi-Pyrénées	44	1	0	8^c^	0	1	0	54^b^
PACA	22	3	0	2	1	0	0	28
Bourgogne	2	1	0	0	0	0	0	3
Pooled areas	83	13	3	18	1	1	0	119

Legend:

*3 digits*: First position: list 1 (hospital department); Second position: list 2 (AIF); Third position: list 3 (Facebook network).

*Presence in the list*: notified as 1; *absence in the list*: notified as 0.

Examples:

^*a*^*Ichthyoses cases reported in list 1 but not reported in lists 2 and 3.*

^*b*^*In Midi Pyrénées area, 54 cases were reported in at least one of the 3 lists.*

^*c*^*Among these 54 patients, 8 patients were recorded in list 1 and in list 2 but not in list 3.*

### Estimation of the number of patients

#### ***Global estimation (pooled-areas level)***

Among all log-linear models considered (Table 2), the independent sources model with 3 covariates effects was selected: it assumes that the 3 sources are independent, and that the capture probabilities depend on each source and on the 3 individual covariates (area, age and severity grade). Estimation was based on data collected from 4 areas (Aquitaine, Midi-Pyrénées, PACA and Pays de Loire), Bourgogne area being excluded because of non-valid estimations due to very low counts. This model gave a global estimation of 191 patients (95% CI [157–253]) for the 4 selected areas.

**Table 2:** Estimations of number of cases from log-linear models including covariates (based on data of four areas, Bourgogne data excluded)

	Weighted AIC*	**Estimated numbers of cases**	**95% CI**
Independent sources model	<.001	184	[150–251]
Independent sources model with age effect	<.001	181	[148–247]
Independent sources model with area effect	<.001	174	[139–260]
Independent sources model with severity effect	<.001	208	[157–324]
Independent sources model with age and area effects	<.001	171	[141–237]
Independent sources model with age and severity effects	<.001	204	[158–299]
Independent sources model with area and severity effects	0.047	194	[154–274]
Independent sources model with three covariates effects	0.951	191	[157–253]

*Weighted AIC: weighted Akaike Information Criteria representing the weight of evidence in favor of a particular model among a set of several models.

#### ***Estimation according to individual covariates***

From this model, we also estimated the number of ichthyosis patients by age group (Table 3): 58 patients under 15 years old (95% CI [47–79]) and 133 patients aged 15 and older (95% CI [102–197]), deducing that 70% of estimated cases were 15 years and older. For each age group, the associated probability of appearing in at least one list was estimated by the ratio recorded/estimated number of patients: 0.72 for patients under 15 years old and 0.56 for patients aged 15 and older. Similarly, we obtained estimations according to the grade of severity. There were more estimated cases with mild disease than with severe disease: 79 (95% CI [55–141]) *vs.* 11 (95% CI [10–17]). The probability of appearing in at least one list increased with disease severity: it was estimated that 95% of very severe patients (grade 4) were detected in the study *vs.* only half of mild patients (grade1).

**Table 3:** Estimated numbers of patients and estimated capture probabilities by individual covariates

**Individual covariate**	**Numbers of recorded patients**	Estimated number of patients^*^	**95% CI**	Estimated capture probability^*^
Age				
Under 15 years	42	58	[49–79]	72%
Older than 15 years	74	133	[102–197]	56%
Severity grade				
Grade 1 (mild)	40	79	[55–141]	50%
Grade 2 (moderate)	40	67	[51–107]	59%
Grade 3 (severe)	26	34	[28–49]	77%
Grade 4 (very severe)	10	11	[10–17]	95%

^*^Estimation obtained from the best log-linear model (independent sources model with three covariates effects).

#### ***Estimation for each area***

The estimated number of ichthyosis patients for each area is presented in Table 4. Bourgogne data were excluded from analysis but can be included in models without covariate. When comparing estimates from these models on 4 or 5 areas, we deduced that the estimated number of patients in Bourgogne was around 9 (no confidence interval available with this *ad hoc* approach).

**Table 4:** Estimated number of patients and estimated prevalence by area

**Area**	**Numbers of recorded patients**	**Estimated number of patients**^**b**^	**95% CI**	**Population (number of residents)**^**c**^	**Estimated prevalence**^**d**^	**95% CI**
Aquitaine	14	30	[19–58]	3 177 625	9.4	[6.0–18.3]
Pays de Loire	20	35	[26–60]	3 510 170	10.0	[7.4–17.1]
Midi-Pyrénées	54	60	[56–77]	2 838 228	21.1	[19.7–27.1]
PACA	28	66	[41–134]	4 882 913	13.5	[8.4–27.4]
4 pooled areas^a^	116	191	[157–253]	14 408 936	13.3	[10.9–17.6]

^a^Data from Bourgogne were not taken into account in this analysis.

^b^Estimation obtained from the best log-linear model (independent sources model with three covariates effects).

^c^Provided by the French 2008 census.

^d^Per million people.

### Estimated prevalence of ichthyosis

The estimated regional prevalence of ichthyosis (95% CI) is presented in Table 4. Estimated prevalence differed by area. The global prevalence of ichthyosis based upon data of the 4-pooled areas was estimated at 13.3 per million people (/M) (95% CI, [10.9–17.6]), which can be considered as national prevalence. When considering prevalence by age group, the prevalence of patients under 15 years old and patients aged 15 and older were estimated at 23/M (95% CI [19–31]) and 11/M (95% CI [9–17]), respectively.

### Estimated prevalence of the different clinical forms of ichthyosis

The diagnosis of the clinical form of ichthyosis was performed by the clinician in charge for 85.7% (102/119) of patients. For the remaining 17 patients (registered in L2 and L3, without overlapping with L1), inclusion criteria were encountered (no patient without ichthyosis, no very mild forms) and the clinical form was unequivocally determined by the expert board. Estimated prevalence for each clinical form is presented in Table 5.

**Table 5:** Estimated prevalence of the different clinical forms of ichthyosis

**Clinical forms of ichthyosis**	Number of recorded patients (percentage)*	**Estimated prevalence per million people (95% confidence interval)**
**All clinical forms**	116	13.3 [10.9 – 17.6]
**Non syndromic ichthyoses***(ichthyosis vulgaris excluded)*	99 (85.3)	11.3 [9.3 – 15.0]
ARCI	61 (52.6)	7.0 [5.7 – 9.2]
*Major variants:*		
Lamellar ichthyosis	39 (33.6)	4.5 [3.7 – 5.9]
Congenital ichthyosiform erythroderma	17 (14.7)	1.9 [1.6 – 2.6]
*Minor variants:*		
Bathing suit ichthyosis	4 (3.4)	0.46 [0.38 – 0.61]
Self healing collodion baby	1 (0.9)	0.11 [0.09 – 0.15]
X-linked ichthyosis	21 (18.1)	2.4 [2.0 – 3.2]
Keratinopathic ichthyosis	10 (8.6)	1.1 [0.9 – 1.5]
Other forms	7 (6.0)	0.80 [0.66 – 1.06]
Erythrokeratodermia variabilis	4 (3.4)	0.46 [0.38 – 0.61]
Peeling Skin Disease	1 (0.9)	0.11 [0.09 – 0.15]
KLICK syndrome	2 (1.7)	0.23 [0.19 – 0.30]
**Syndromic ichthyoses**	17 (14.7)	1.9 [1.6 – 2.6]
Syndromic X-linked ichthyosis	1 (0.9)	0.11 [0.09 – 0.15]
Conradi – Hünermann – Happle syndrome	2 (1.7)	0.23 [0.19 – 0.30]
Netherton syndrome	7 (6.0)	0.80 [0.66 – 1.06]
Trichothiodystrophy	2 (1.7)	0.23 [0.19 – 0.30]
Sjögren – Larsson syndrome	1 (0.9)	0.11 [0.09 – 0.15]
KID syndrome	3 (2.6)	0.34 [0.28 – 0.45]
Neutral lipid storage disease	1 (0.9)	0.11 [0.09 – 0.15]

*Bourgogne area excluded (3 patients).

More than 85% of the patients had a non-syndromic form of ichthyosis, with two-thirds diagnosed as Autosomal Recessive Congenital Ichthyosis (ARCI). Prevalence of ARCI was estimated at 7/M (95% CI [5.7–9.2]) with a ratio Lamellar Ichthyosis (LI)/Congenital Ichthyosiform Erythroderma (CIE) of 2.4. When considering prevalence of ARCI by age group, the prevalence of patients under 15 years old and aged 15 and older were estimated at 12.1/M (95% CI [10.2 – 16.5] and 5.9/M (95% CI [4.5 – 8.7]), respectively. Prevalence of recessive X-linked Ichthyosis (XLI) was estimated to 2.4/M, i.e. 4.9 cases per million males (CI 95% [4.1 – 6.6]), according to a ratio female/male: 51.4%/48.6%, provided by the French 2008 census). Prevalence of keratinopathic forms was even lower: 1.1/M (95% CI [0.9–1.5]).

Less than 15% of patients had syndromic ichthyosis with an estimated prevalence of 1.9/M (95% CI [1.6–2.6]). Netherton syndrome was the most frequent form, with 0.80/M (95% CI [0.66–1.06]).

## Discussion

This is the first study investigating prevalence of inherited ichthyosis (excluding very mild forms) in France. This prevalence was estimated at 13.3/M (95% CI, [10.9–17.6]), with more than half of the patients suffering from ARCI.

Capture-recapture method was appropriate for estimating prevalence of rare diseases. Indeed, cross-sectional studies are the main alternative to estimate prevalence but may fail in case of rare disease because, even in large samples, it is possible to detect no case [27]. Requirements of the capture-recapture method were respected [18]. The target population was closed (no entry or loss of patients during the study period); individuals have been correctly recorded; there was no matching error; any individual of the target population had a strictly positive probability to appear on any list.

The choice of our 3 lists was based on the followings points: patients are often seen even once in expert centres for rare skin disease (L1). The association AIF (L2) is the only patient support group in France and registers a significant number of patients (approximately 250 members). The choice of a social network (L3) was based on patients’ enthusiasm for searching information or communicating *via* internet (more than 100 members). Recruitment via L2 and L3 (17 non-hospital patients: 14.3%) was not negligible which confirmed the appropriateness of the capture-recapture method and the choice of the lists. The choice of the 5 areas was based on the presence of a reference/competence centre. These areas can be considered as representative of France since they are scattered throughout the country and cover more than 20% of the population.

From a statistical point of view, sparse and unbalanced data induced some difficulties. Indeed it was not possible to include the clinical form in our selection model procedure because the statistical procedure implemented by SAS did not provide valid estimates due to computational difficulties. These statistical difficulties were solved by implementing *ad hoc* approaches. Other statistical approaches which require some specific theoretical developments are possible and could be proposed in future studies. To complete our statistical analysis, the pseudo-R^2^ proposed by Mc Fadden [28] was calculated: 74% of data variability was explained by the selected model, highlighting its good fitness and its explanatory power.

One limitation of the study could be the use of a self-assessment severity scoring by patients from L2 and L3 who could not be seen by the clinicians involved in the study. Nevertheless, all diagnoses were unequivocally determined by the expert board. In addition, our study did not provide an estimation of the prevalence of the entire ichthyosis population. This could be achieved in the future by the creation of well-designed register involving also private physicians.

In the literature, only a few studies have specifically focused on ichthyosis’s prevalence. Earliest studies were conducted outside Europe (Saudi Arabia and Tunisia) and concluded to prevalence which seems different than European one because of a high level of consanguinity [9-12]. In Europe, two recent Spanish studies have dealt with ichthyosis prevalence [13,14]. Contrary to our study, only ARCI were concerned. The first one found a prevalence of ARCI consistent with ours, although slightly higher (8.2/M) but the method used for estimation was not clearly described [13]. The second one was an epidemiological study (144 patients) that used the same capture-recapture method [14]. ARCI’s prevalence was almost identical to ours, with a similar proportion of LI/CIE/minor variants: 7.2/M (CI 95% [5.7 – 9.7]; 62.5/30.6/7%) for the Spanish estimation *vs.* 7/M (CI 95% [5.7 – 9.2]; 64/28/8%) for our study. In addition, estimates of ARCI’s prevalence in the Spanish population depended on the age group (the younger the age group, the higher the prevalence) with an ARCI’s prevalence of 16.2/M (CI 95% [13.3 – 23.0]) for the patients younger than 10 years. This age effect was also found in our estimates (23/M (95% CI [19–31]) for patients younger than 15 years old (12.1/M (95% CI [10.2 – 16.5]) when only considering ARCI). In the United States, incidence of ichthyosis during the first year of life (excluding common forms which are not diagnosed at birth) was estimated to 6.7 per 100.000 from large administrative claims databases [15].

With regards to keratinopathic ichthyosis, a recent Japan survey had estimated the number of patients to 55 (CI 95% [35 – 75]) which corresponds to a lower prevalence (0.44/M (CI 95% [0.28 – 0.59])) than ours. Nevertheless, Japanese patients seen only in Dermatology departments were concerned [16]. With regard to XLI, some studies had concluded to a substantial prevalence, possibly excessive, that ranges from 1 case per 10 000 to 1 case per 2 000 males (large ratio of 5:1), depending on the study population and the method of assessment [29-32]. Our XLI prevalence was lower. It is therefore difficult to conclude formally about the real prevalence of XLI. Our prevalence may be underestimated and could mostly concern patients who are suffering from moderate to very severe forms. Mild forms of XLI are probably less bothersome and these patients may not consult a hospital or be members of patient’s association or social network. This is in accordance with the small number of patients (n = 73) diagnosed as XLI during the 10 past years in France using enzymatic blood test (personal data provided by the only French diagnosis centre).

## Conclusion

This is the first French epidemiological study on ichthyosis. It focused on ichthyoses with significant impact on QOL, usually followed in hospital and for which medical, social or research progresses are needed. The obtained results constitute an essential basis for properly sizing the necessary health measures such as access to molecular diagnosis or design of clinical studies, especially in the domain of therapeutics in which there is a true lack.

## Competing interests

The authors declare that they have no competing interest.

## Authors’ contributions

ID designed and coordinated the study, contributed to collecting data, performed the revision and the interpretation of all the clinical data and wrote the manuscript. CC and JD co-designed the study, performed the statistical analyses and co-wrote the manuscript. SP participated in the statistical analyses. SH, CC, AM, LR, PV, LM, SM and SB contributed to collecting data. KE contributed towards collecting data and revising the manuscript. JMH designed and co-coordinated the study, contributed to the acquisition and interpretation of data and co-wrote the manuscript. All authors read and approved the final manuscript.

## References

[B1] Oji V Traupe H Ichthyosis: clinical manifestations and practical treatment options Am J Clin Dermatol 2009 10 351 364 10.2165/11311070-000000000-00000 19824737

[B2] Oji V Tadini G Akiyama M Blanchet Bardon C Bodemer C Bourrat E Coudiere P DiGiovanna JJ Elias P Fischer J Fleckman P Gina M Harper J Hashimoto T Hausser I Hennies HC Hohl D Hovnanian A Ishida-Yamamoto A Jacyk WK Leachman S Leigh I Mazereeuw-Hautier J Milstone L Morice-Picard F Paller AS Richard G Schmuth M Shimizu H Sprecher E Revised nomenclature and classification of inherited ichthyoses: results of the First Ichthyosis Consensus Conference in Sorèze 2009 J Am Acad Dermatol 2010 63 607 641 10.1016/j.jaad.2009.11.020 20643494

[B3] Gånemo A Lindholm C Lindberg M Sjödén PO Vahlquist A Quality of life in adults with congenital ichthyosis J Adv Nurs 2003 44 412 419 10.1046/j.0309-2402.2003.02820.x 14651713

[B4] Gånemo A Sjödén PO Johansson E Vahlquist A Lindberg M Health-related quality of life among patients with ichthyosis Eur J Dermatol 2004 14 61 66 14965800

[B5] Kamalpour L Gammon B Chen KH Veledar E Pavlis M Rice ZP Chen SC Resource utilization and quality of life associated with congenital ichthyoses Pediatr Dermatol 2011 28 512 518 10.1111/j.1525-1470.2011.01432.x 21895756

[B6] Mazereeuw – Hautier J Dreyfus I Barbarot S Serrentino L Bourdon – Lanoy E Ezzedine K Maza A Aujoulat I Le Rhun A Factors influencing quality of life in patients with inherited ichthyosis : a qualitative study using focus groups Br J Dermatol 2012 166 646 648 10.1111/j.1365-2133.2011.10701.x 22014001

[B7] Dreyfus I Bourrat E Maruani A Bessis D Chiavérini C Vabres P Ezzedine K Mazereeuw–Hautier J Factors associated with impaired quality of life in adult patients suffering from inherited ichthyosis Acta Derm Venereolin press 10.2340/00015555-1710 24158261

[B8] Fischer J Autosomal recessive congenital ichthyosis J Invest Dermatol 2009 129 1319 1321 10.1038/jid.2009.57 19434086

[B9] Al-Zayir AA Al-Amro Al-Alakloby OM Primary hereditary ichthyoses in the eastern province of Saudi Arabia Int J Dermatol 2004 43 415 419 10.1111/j.1365-4632.2004.01687.x 15186221

[B10] Al-Zayir AA Al-Amro Al-Alakloby OM Clinico-epidemiological features of primary hereditary ichthyoses in the eastern province of Saudi Arabia Int J Dermatol 2006 45 257 264 10.1111/j.1365-4632.2006.02042.x 16533225

[B11] Al-Amro Al-Akloby OM Al-Zayir AA Clinico-epidemiological features of congenital nonbullous ichthyosiform erythroderma in the eastern province of Saudi Arabia J Eur Acad Dermatol Venereol 2004 18 659 664 10.1111/j.1468-3083.2004.01042.x 15482290

[B12] Kharfi M El Fekih N Ammar D Khaled A Fazaa B Ridha Kamoun M Hereditary Ichthyosis in Tunisia: epidemiological of 60 cases Tunis Med 2008 86 983 986 19213489

[B13] Rodríguez-Pazos L Ginarte M Fachal L Toribio J Carracedo A Vega A Analysis of TGM1, ALOX12B, ALOXE3, NIPAL4 and CYP4F22 in autosomal recessive congenital ichthyosis from Galicia (NW Spain): evidence of founder effects Br J Dermatol 2011 165 906 911 10.1111/j.1365-2133.2011.10454.x 21668430

[B14] Hernández-Martín A Garcia-Doval I Aranegui B de Unamuno P Rodríguez-Pazos L González-Enseñat MA Vicente A Martín-Santiago A Garcia-Bravo B Feito M Baselga E Círia S de Lucas R Ginarte M González-Sarmiento R Torrelo A Prevalence of autosomal recessive congenital ichthyosis: a population-based study using the capture-recapture method in Spain J Am Acad Dermatol 2012 67 240 244 10.1016/j.jaad.2011.07.033 22000705

[B15] Milstone L Miller K Haberman M Dickens J Incidence of moderate to severe ichthyosis in the United States Arch Dermatol 2012 148 1080 1081 10.1001/archdermatol.2012.1702 22986871

[B16] Kurosawa M Takagi A Tamakoshi A Kawamura T Inaba Y Yokoyama K Kitajima Y Aoyama Y Iwatsuki K Ikeda S Epidemiology and clinical characteristics of bullous ichthyosisform erythroderma (keratinolytic ichthyosis) in Japan: results from a nationwide survey J Am Acad Dermatol 2013 68 278 283 10.1016/j.jaad.2012.06.044 23182068

[B17] Le site santé du Ministère des Affaires sociales et de la Santé http://www.sante.gouv.fr/IMG/pdf/Plan_national_maladies_rares_2011-2014.pdf 23923514

[B18] International Working Group for Disease Monitoring and Forecasting (IWGDMF) Capture-recapture and multiple-record systems estimation I: history and theoretical development Am J Epidemiol 1995 142 1047 1058 7485050

[B19] International Working Group for Disease Monitoring and Forecasting (IWGDMF) Capture-recapture and multiple-record systems estimation II: application in human diseases Am J Epidemiol 1995 142 1059 1068 7485051

[B20] Chao A Tsay PK Lin SH Shau WY Chao DY The applications of capture-recapture models to epidemiological data Stat Med 2001 20 3123 3157 10.1002/sim.996 11590637

[B21] Hook EB Albright SG Cross PK Use of Bernoulli census and log-linear methods for estimating the prevalence of spina bifida in livebirths and the completeness of vital record reports in New York State Am J Epidemiol 1980 112 750 758 7006383 10.1093/oxfordjournals.aje.a113047

[B22] Hook EB Regal RR Recommendations for presentation and evaluation of capture-recapture estimates in epidemiology J Clin Epidemiol 1999 52 917 926 10513754 10.1016/s0895-4356(99)00060-8

[B23] Hook EB Regal RR On the need for a 16th and 17th recommendations for capture-recapture analysis J Clin Epidemiol 2000 53 1275 1277 10.1016/S0895-4356(00)00247-X 11188846

[B24] Dupuis JA Schwarz CJ A Bayesian approach to the multistate Jolly–Seber capture–recapture model Biometrics 2007 63 1015 1022 10.1111/j.1541-0420.2007.00815.x 17501941

[B25] Tilling K Sterne J Capture-recapture models including covariate effects Am J Epidemiol 1999 149 392 400 10.1093/oxfordjournals.aje.a009825 10025483

[B26] Burnham KP Anderson DR Model selection and multimodel inference: a practical information-theoretic approach 2002 New York: Springer

[B27] Mann CJ Cohort, cross sectional and case–control studies: research design II Emerg Med J 2003 20 54 60 10.1136/emj.20.1.54 12533370 PMC1726024

[B28] Shtatland ES Moore S Barton MB Why we need R^2^ measure of fit (and not only one) PROC LOGISTIC and PROC GENMOD. SUGI 2000 Proceeding 2000 Cary, NC: SAS Institute, Inc 1338 1343 SUGI 2000 Proceeding

[B29] Lykkesfeldt G Nielsen MD Lykkesfeldt AE Placental steroid sulfatase deficiency: biochemical diagnosis and clinical review Obstet Gynecol 1984 64 49 54 10.1097/00006250-198409001-00013 6234482

[B30] Wells RS Kerr CB Clinical features of autosomal dominant and sex-linked ichthyosis in an English population Br Med J 1966 1 947 950 10.1136/bmj.1.5493.947 20790920 PMC1844863

[B31] Ingordo V D’Andria G Gentile C Decuzzi M Mascia E Naldi L Frequency of X-linked ichthyosis in coastal southern Italy: a study on a representative sample of the male population Dermatology 2003 207 148 150 10.1159/000071784 12920363

[B32] Sakura N Nichimura SI Matsumoto T Ohsaki M Frequency of steroid sulfatase deficiency in Hiroshima Acta Paediatr Jpn 1998 40 63 64 9583203 10.1111/j.1442-200x.1998.tb01404.x

